# Seizure outcome-related factors in autoimmune encephalitis: A systematic review and meta-analysis

**DOI:** 10.3389/fneur.2022.991043

**Published:** 2022-11-03

**Authors:** Peijuan Luo, Rui Zhong, Qingling Chen, Weihong Lin

**Affiliations:** ^1^Department of Neurology, The First Hospital of Jilin University, Changchun, China; ^2^Department of Hepatology, Second People's Clinical College of Tianjin Medical University, Tianjin, China

**Keywords:** autoimmune encephalitis, seizure outcome, persistent seizures, risk factors, meta-analysis

## Abstract

**Background:**

Identifying the predictors for seizure outcome in autoimmune encephalitis (AE) and investigating how to prevent persistent seizures would have major clinical benefits effectively. Thus, we aimed to perform a systematic review and meta-analysis to examine seizure outcome-related factors in AE patients.

**Methods:**

PubMed and EMBASE were systematically searched from inception to 10 June 2022 for studies investigating seizure outcome-related factors in AE. The pooled effect estimates, including standardized mean differences (SMDs) and odds ratios (ORs) with 95% confidence intervals (CIs), were calculated to estimate the effect of each included factor on the seizure outcome.

**Results:**

A total of 10 studies were included in the meta-analysis. Our pooled results of this meta-analysis showed that five factors were found to increase the risk of persistent seizures in AE patients, including onset with seizures (OR = 2.106, 95% CI = 1.262–3.514, *p* = 0.004), status epilepticus (OR = 3.017, 95% CI = 1.995–4.563, *p* < 0.001), EEG abnormalities (OR = 1.581, 95% CI = 1.016–2.46, *p* = 0.042), MRI abnormalities (OR = 1.554, 95% CI = 1.044–2.283, *p* = 0.03), and longer time from clinical onset to immunotherapy (SMD = 1.887, 95% CI = 0.598–3.156, *p* = 0.004).

**Conclusion:**

Our meta-analysis indicated that onset with seizures, status epilepticus, EEG abnormalities, MRI abnormalities, and longer time from clinical onset to immunotherapy were risk factors for persistent seizures in AE patients.

## Introduction

Autoimmune encephalitis (AE) comprises a group of non-infectious inflammatory brain diseases mediated by antibodies that attack surface receptors and ion channels on neurological tissues (1–4). Acute symptomatic seizures are a common manifestation in the acute phase of AE (5, 6). Our recent study reported that 86% of AE patients experienced acute seizures (6). In a cohort of anti-NMDAR encephalitis patients, 80.7% of patients reported seizures at the acute stage (7). Most AE patients could reach seizure-free faster after initial immunotherapy, and antiseizure medications (ASMs) should be considered as add-on treatment (8). The efficacy of ASM treatment alone was low in AE patients (9).

The risk of experiencing persistent seizures or developing chronic epilepsy after resolved AE has been uncertain. A recent prospective cohort study reported that 9.3% of patients experienced seizure recurrence and 3.1% developed chronic epilepsy after the acute phase of AE (10). However, Zhang and his colleagues reported that 37.2% of AE patients developed persistent seizures after discharge (11). The early identification of AE patients at high risk of experiencing persistent seizures would provide insights into intervention and new therapy approaches. Thus, identifying the predictors for seizure outcome and investigating how to prevent persistent seizures effectively would have major clinical benefits. Previous literature investigating such predictors has examined sex, status epilepticus, EEG abnormalities, time from clinical onset to immunotherapy, and antibody titer (6, 7, 10, 12). We recently reported that abnormal EEG findings and delayed immunotherapy increased the risk of persistent seizures (6). Onset with seizures may also contribute to a poor seizure outcome (12). Meng and her colleagues reported that AE patients with status epilepticus were prone to having a higher risk of developing persistent seizures (11). However, some conclusions seem to be conflicting, confusing our knowledge on this topic.

To date, no meta-analysis on seizure outcome-related factors in AE has been performed. Thus, we aimed to perform a systematic review and meta-analysis to investigate the predictors of persistent seizures or chronic epilepsy in AE patients.

## Methods

This systematic review and meta-analysis were conducted according to the recommendations by the Meta-Analysis of Observational Studies in Epidemiology Group, the Preferred Reporting Items for Systematic Reviews and Meta-Analyses (PRISMA) 2009 guidelines (13–15).

### Search strategy

PubMed and EMBASE were systematically searched by two independent reviewers (P. L and Q. C) from inception to 10 June 2022 for studies investigating seizure outcome-related factors in autoimmune encephalitis (AE). We used the following search terms: (“autoimmune encephalitis” OR “anti-NMDAR encephalitis” OR “anti-GABABR encephalitis” OR “anti-LGI1 encephalitis”) AND (“epilepsy” OR “seizure”). References of original studies, relevant reviews, and meta-analyses were hand-searched for further supplementation.

### Selection criteria

Published literature was included if they simultaneously met the following criteria: (1) all involved AE patients were grouped according to seizure outcome; (2) the diagnosis of AE patients was based on definitive diagnostic criteria; (3) sufficient data on predictors for seizure outcome studied in this meta-analysis were reported; and (4) retrospective or prospective cohort studies published in English. Articles were excluded for the following reasons: (1) reviews, meta-analyses, letters, case reports, and conference abstracts; (2) incomplete data; and (3) duplicated articles. If two published studies were based on the same cohort, we chose the study with larger sample size. Divergences in the study selection process were resolved through a discussion in the third part.

### Outcome and potential factors

Seizure outcome was assessed based on whether the patient experienced persistent seizures and developed epilepsy after the acute phase (5, 12). Seizure remission is defined as a period of uninterrupted seizure freedom lasting 6 months or longer (16). According to the seizure outcome, AE patients were divided into a persistent seizure/epilepsy group and a seizure remission/seizure-free group. The choice of seizure outcome-related factors was based on physicians' experience and literature. We only analyzed the factors with a relatively large population (described by at least three studies) to lower the error of estimates. The presence of epileptiform discharges (focal or generalized spike waves) on an EEG was defined as abnormalities (6). Hyperintensity on T2-weighted imaging (T2WI) and fluid-attenuated inversion recovery (FLAIR) imaging and hypointensity on T1-weighted imaging (T1WI) were defined as abnormal brain MRI.

### Data extraction and quality assessment

The following data were extracted using a predesigned standard form: name of the first author, publication year, country, sample size, study design, follow-up time, mean age, gender proportion (female), antibody types, and original data. The quality of each selected study was assessed using the Newcastle–Ottawa Scale (NOS) guidelines (17), with an NOS score ≥ 7 indicating high quality. All discrepancies were discussed until a consensus was achieved.

### Statistical analyses

The pooled effect estimates, including standardized mean differences (SMDs) and odds ratios (ORs) with 95% confidence intervals (CIs), were calculated to estimate the effect of each included factor on the seizure outcome. We measured heterogeneity using the I2 statistic and Q statistic (18). I^2^ > 50% and *P* < 0.05 suggested significant heterogeneity across the included studies; hence, a random-effect model was subsequently employed. A fixed-effect model was used when heterogeneity was not significant. The source of heterogeneity was explored *via* sensitivity analysis if significant heterogeneity existed. Publication bias was assessed using funnel plots only when a factor was reported by ≥10 studies. Statistical significance was *p* < 0.05. All statistical analyses were performed using STATA version 12.0 (StataCorp, Texas).

## Results

### Study selection and characteristics

**Figure 1:**
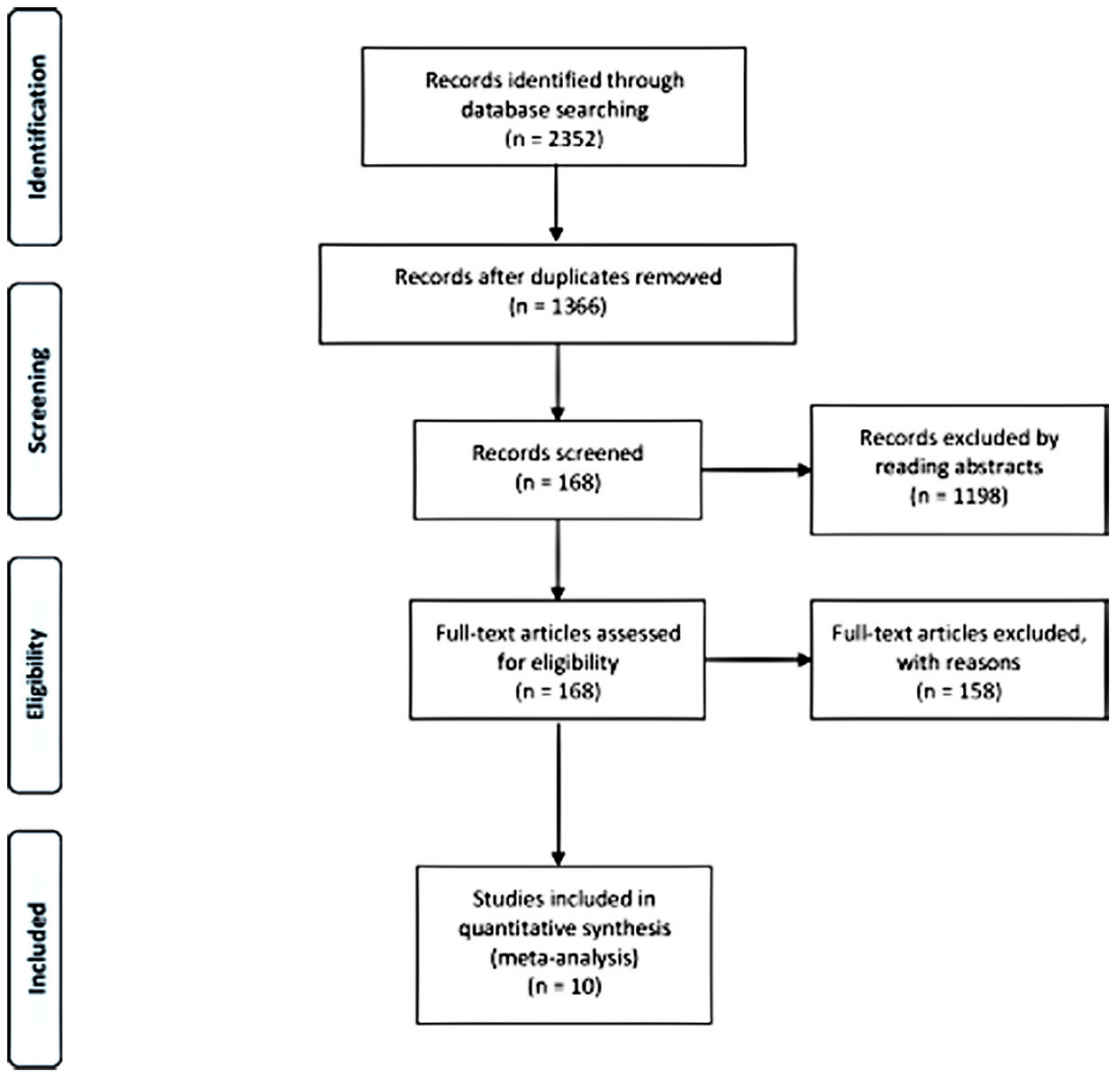
Flowchart of the study selection process.

A total of 2,352 articles were initially identified after the literature search. In addition, 986 duplicated articles were removed. Then, 1,198 articles were excluded after screening the titles and abstracts, leaving 168 articles with full text available. Eventually, 10 studies were included in the meta-analysis (6, 11, 12, 16, 19–24) (Figure 1). Nine were retrospective cohort studies, and one was a prospective cohort study; the follow-up time ranged from 7.16 to 48 months, and the sample size ranged from 19 to 111 individuals. The characteristics of each included article are shown in Table 1. The 10 studies showed high quality (≥7 points in NOS).

**Table 1:** Characteristics of all included studies in our meta-analysis.

**References**	**Country**	**Sample size**	**Study design**	**Follow up** **(months)**	**Mean age** **(Years)**	**Female (%)**	**Antibodies types**	**Factors reported**	**NOS scores**
Guery et al. (19)	France	39	Retrospective cohort	42	63	14 (36%)	LGI1	F1, F4, F5, F6, F7	8
Zhong et al. (6)	China	86	Retrospective cohort	21	48	42 (48.8%)	NMDAR, LGI1, GABABR	F1, F2, F3, F4, F5, F6, F7	8
Gifreu et al. (20)	Spain	19	Retrospective cohort	7.16	52.79	9 (47.37)	GAD, NMDAR, LGI1	F1, F2, F4, F6, F7	7
Chen et al. (21)	China	111	Retrospective cohort	> 6	36.8	61 (55%)	NMDAR, LGI1,GABABR,GAD-65, Caspr2	F1, F2, F3, F4, F6, F7	7
Lin et al. (22)	China	70	Retrospective cohort	>24	60	21 (30%)	LGI1	F1, F2, F4, F5, F6	8
Wang et al. (12)	China	56	Retrospective cohort	>12	_	27 (48.2%)	NMDAR, LGI1, GABABR	F1, F2, F3, F4, F5, F6	8
Shen et al. (16)	China	80	Prospective cohort	30.5	36.4	36 (45.0%)	NMDAR, LGI1, GABABR	F1, F2, F4, F5, F6	8
Zhang et al. (11)	China	52	Retrospective cohort	30	46	23 (44.2)	NMDAR, LGI1, GABABR	F1, F2, F3, F4, F5, F6	7
Qu et al. (23)	China	62	Retrospective cohort	48	6.5	31 (50.%)	NMDAR	F1, F2, F3, F4, F5, F6, F7	7
Casciato et al. (24)	Italy	33	Retrospective cohort	19	61.2	14 (42.4%)	NMDAR, LGI1,GAD-65, Caspr2, SOX1	F5, F6	7

Risk factors: F1, age at onset; F2, gender (female); F3, onset with seizures; F4, status epilepticus; F5, EEG abnormality; F6, MRI abnormality; F7, time from clinical onset to immunotherapy.

### Seizure outcome-related factors

Data were extracted in AE patients with or without persistent seizures from 10 studies. Here, we only presented the factors with a relatively large population (reported in at least three studies) to lower the error of estimates. Thus, seizure outcome-related factors in the meta-analysis included age at onset, sex proportion (female), onset with seizures, status epilepticus, EEG abnormalities, MRI abnormalities, and time from clinical onset to immunotherapy (Table 2).

**Table 2:** Pooled analysis of each included risk factor for persistent seizures in this meta-analysis.

**Risk factors**	**Number of included studies**	**Sample size**	**Pooled effects**			**Heterogeneity**		**Analysis model**
			**OR/SMD**	**95% CI**	***P*** **–value**	**I^2^, %**	***P*** **–value**	
Age at onset (years)	9	575	0.119	0.174–0.413	0.426	53.40%	0.028	Random
Female	8	536	1.13	0.762–1.675	0.543	0%	0.927	Fixed
Onset with seizures	5	367	2.106	1.262–3.514	0.004	8.90%	0.355	Fixed
Status epilepticus	9	575	3.017	1.995–4.563	< 0.001	0%	0.553	Fixed
EEG abnormality	9	497	1.581	1.016–2.46	0.042	46.10%	0.062	Fixed
MRI abnormality	10	608	1.544	1.044–2.283	0.03	0%	0.823	Fixed
Time from clinical onset to immunotherapy	5	317	1.887	0.598–3.156	0.004	93.80%	< 0.001	Random

### Age at onset

A total of nine studies representing 575 participants were about the age at onset. Meta-analysis results suggested no statistically significant difference in terms of age at onset between patients with and without persistent seizures (SMD = 0.119, 95% CI = −0.174–0.413, *p* = 0.426). A random-effect model was used due to the significant heterogeneity (I^2^ = 53.40%, *p* = 0.028). The sensitivity analysis results suggested that heterogeneity was not reduced (I^2^ change > 30%) when one single study was removed each time.

### Gender proportion (female)

A total of eight studies involving 536 participants were about gender proportion. Meta-analysis results showed no statistically significant difference in sex proportion between the persistent seizure group and the seizure remission group (OR = 1.13, 95% CI = 0.762–1.675, *p* = 0.543). No statistical heterogeneity was detected among these studies, so a fixed-effect model was employed (I^2^ = 0%, *p* = 0.927).

### Onset with seizures

A total of five studies involving 367 participants were about the onset with seizures. Meta-analysis results showed that onset with seizures increased the risk of persistent seizures in AE patients (OR = 2.106, 95% CI = 1.262–3.514, *p* = 0.004). No statistical heterogeneity was detected among these studies, so a fixed-effect model was employed (I^2^ = 8.9%, *p* = 0.355).

### Status epilepticus

A total of nine studies involving 575 participants were about status epilepticus. Meta-analysis results showed that the risk of persistent seizures significantly increased in AE patients who experienced status epilepticus at the acute phase (OR = 3.017, 95% CI = 1.995–4.563, *p* < 0.001). No statistical heterogeneity was detected among these studies, so a fixed-effect model was employed (I^2^ = 0%, *p* = 0.553).

### EEG abnormalities

A total of nine studies involving 497 participants were about EEG abnormalities. Meta-analysis results showed that EEG abnormalities increased the risk of persistent seizures in AE patients (OR = 1.581, 95% CI = 1.016–2.46, *p* = 0.042). No statistical heterogeneity was detected among these studies, so a fixed-effect model was employed (I^2^ = 46.1%, *p* = 0.062).

### MRI abnormalities

**Figure 2:**
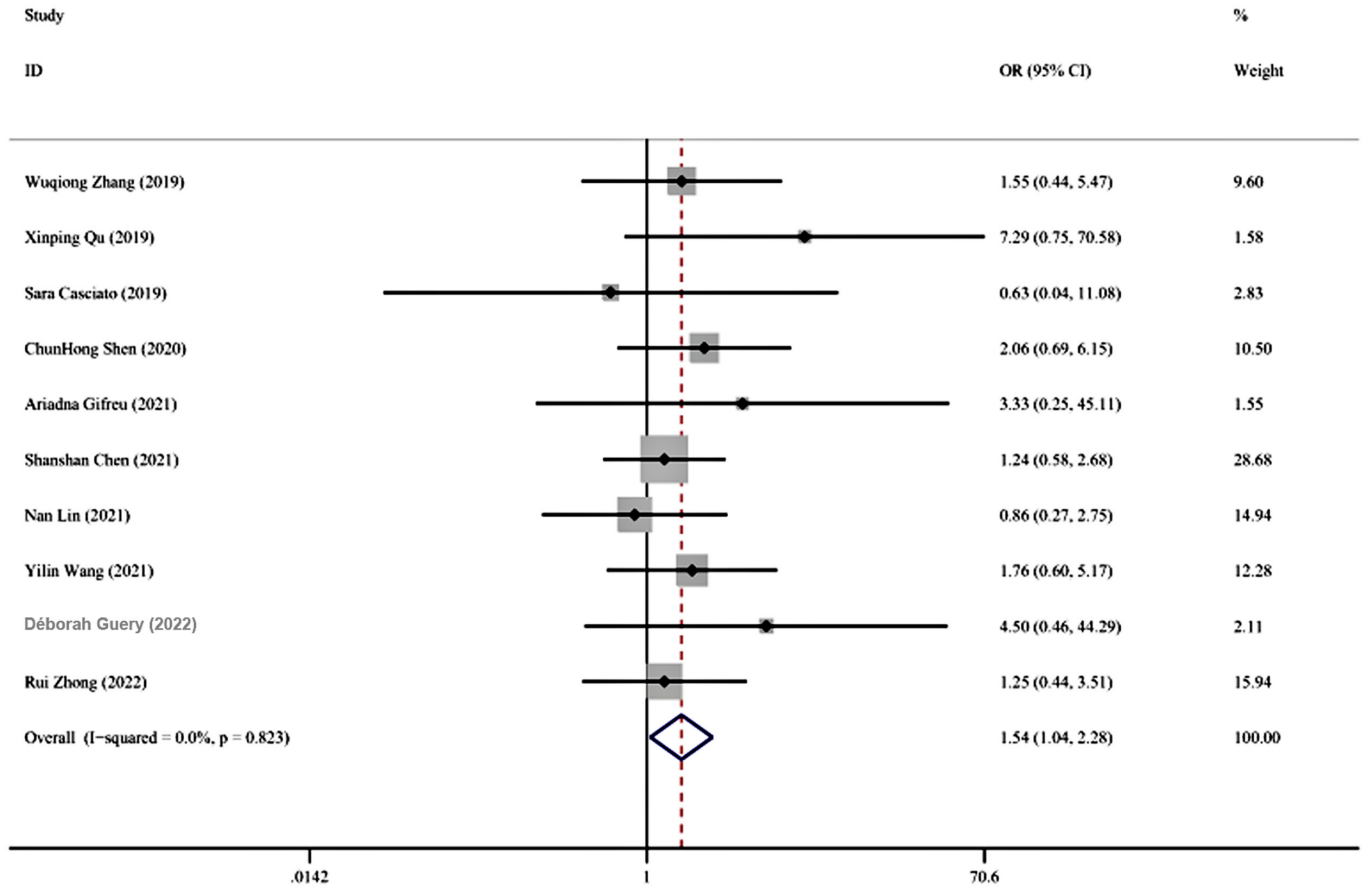
Forest plot of the association between MRI abnormalities and persistent seizures.

**Figure 3:**
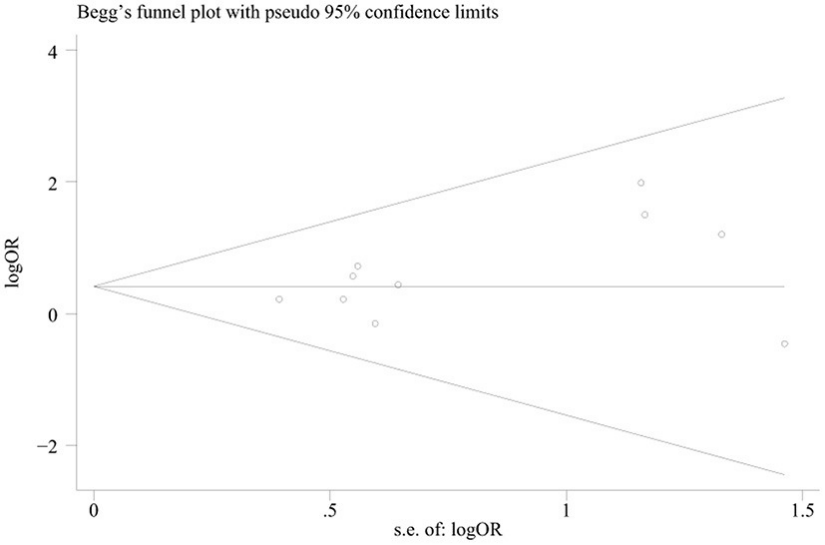
Funnel plot suggests no obvious publication bias.

A total of 10 studies involving 608 participants were about MRI abnormalities. Meta-analysis results showed that MRI abnormalities were associated with an increased risk of persistent seizures in AE patients (OR = 1.544, 95% CI = 1.044–2.283, *p* = 0.03) (Figure 2). No statistical heterogeneity was detected among these studies, so a fixed-effect model was employed (I^2^ = 0%, *p* = 0.823). The funnel plots indicated that no publication bias existed (Figure 3).

### Time from clinical onset to immunotherapy

A total of five studies involving 317 participants were about the time from clinical onset to immunotherapy. Meta-analysis results showed that patients in the persistent seizure group were prone to experience a longer time from clinical onset to immunotherapy than those in the seizure remission group (SMD = 1.887, 95% CI = 0.598–3.156, *p* = 0.004). A random-effect model was used due to the significant heterogeneity (I^2^ = 93.8%, *p* < 0.001). Additionally, the sensitivity analysis did not find the source of heterogeneity.

## Discussion

This systematic review involved extensive analysis and shed new light on early predictors of persistent seizures in AE. Seven factors were available for meta-analysis. We found that onset with seizures, status epilepticus, EEG abnormalities, MRI abnormalities, and longer time from clinical onset to immunotherapy was associated with an increased risk of persistent seizures in AE patients. In contrast, there was no evidence that age at onset and sex affected seizure outcomes.

Prior literature has reported that onset with seizures and status epilepticus occurrence were associated with a poor seizure outcome (12, 21). However, some researchers hold the opposite findings (6, 16). Consistently, we found in this meta-analysis that onset with seizures and status epilepticus had adverse effects on seizure outcomes and increased the risk of developing persistent seizures. Similarly, status epilepticus has been identified as an independent predictor of acquired epilepsy among stroke survivors (25). Additionally, it has been reported that timely termination of status epilepticus leads to a good seizure outcome (26). The complications of status epilepticus, such as severe pneumonia and ICU admission, have been associated with poor outcomes (27). The mechanism for the association of status epilepticus with seizure outcome remains elusive in these AE patients. Prior evidence has shown that autoimmune status epilepticus is refractory to ASMs (28).

Interictal epileptiform discharges (IEDs) were an important risk factor for poor seizure outcomes in patients with anti-NMDAR, anti-LGI1, and anti-GABABR encephalitis, particularly in those with anti-NMDAR encephalitis (16). However, another recent study showed that EEG abnormalities were not seizure outcome-related factors in AE patients (12). The inconsistent results of the EEG findings across previous studies may be attributed to a difference in the time-point of the examination and basic characteristics. Thus, it will be necessary to underscore the importance of the persistence of IEDs after the resolution of the acute phase. In the early stage of AE, EEG results are usually normal and gradually present abnormalities with the progression of the disease. EEG abnormalities after resolved encephalitis may be rare in AE patients treated with immunotherapy. Furthermore, different onset symptoms may be responsible for different time points of EEG examination. For example, some patients may have onset with seizures, thereby contributing to a more complete presentation of abnormalities on EEG at the early stage.

However, our meta-analysis showed that abnormal EEG findings had a negative impact on seizure outcomes, and there was no significant heterogeneity among the included studies. The exact mechanism remains uncertain. One of the possible mechanisms was intrinsic disease severity. Evidence also suggests that EEG abnormalities may predict an increased risk of seizure recurrence and the development of drug-resistant epilepsy in newly diagnosed epilepsy (29, 30). According to the literature, brain MRI with hippocampal atrophy is a significant predisposing factor associated with the risk of developing epilepsy in AE patients (20). In another cohort study, early brain MRI abnormalities significantly contributed to chronic epilepsy in AE (21). However, the main regions involved were the parietal or frontal lobe, not the temporal lobe (8, 21, 31, 32). Temporal lesions were the more common in AE patients (21). Similarly, our analysis confirmed this association between MRI abnormalities and seizure outcomes.

According to the literature, the administration of immunotherapy was significantly associated with seizure outcome, and delay of immunotherapy initiation was also related to the development of drug-resistant epilepsy (8, 16, 33, 34). A delay in diagnosis or the initiation of immunotherapy is partly due to some patients' atypical and insidious symptoms in some patients. Seizure remission can be reached faster and more frequently after the initiation of immunotherapy (8). Our pooled analysis also indicated that a long time for immunotherapy was a risk factor for persistent seizures. This may be because delayed immunotherapy is related to an increased risk of aggravation of the autoimmune process in the brain, which contributes to the development of epilepsy. Thus, in clinical practice, we should prioritize immunotherapy to control acute seizures as soon as possible and improve seizure outcomes.

This meta-analysis had several limitations. First, we could not perform subgroup analysis by antibody type due to the unconformity of original studies and the limited sample size. Second, some potential risk factors for persistent seizures were not analyzed when one or two studies reported them. For, example, Zhong et al. recently reported that a larger number of ASMs was related to an increased risk of persistent seizures (6). Furthermore, high antibody titer may lead to a poor seizure outcome (12). Moreover, the serological status may be associated with seizure outcome, which was not analyzed in our meta-analysis. Because, we found that all included studies were based on patients with antibodies against neuronal cell surface proteins, such as anti-NMDAR, anti-LGI1, and anti-GABA B R encephalitis. The number of patients with seronegative/antibodies against intracellular neuronal proteins was limited. It is an important question whether the kind of MRI abnormalities was associated with seizure outcome in AE. However, the data on the detailed features of MRI abnormalities were unavailable in most included studies. Third, there was significant heterogeneity in age at onset and time from clinical onset to immunotherapy, and the sensitivity analysis did not find the source of heterogeneity. Fourth, a total of 10 studies were included in this meta-analysis. However, certain eligible articles might be missed even though systemic studies were performed. Fifth, seizure outcome was defined differently across studies. We chose persistent seizures as the outcome of this meta-analysis. Sixth, publication bias was not assessed in most meta-analyses due to the limited number of included studies (*n* < 10). Seventh, most studies included in this meta-analysis were conducted in Asia, so our results cannot easily be extended to the whole world population. Finally, we did not describe how many patients were seizure-free without ASMs as this information was not available in these included studies. Identifying how long ASM therapy needs to be maintained in EA patients will be beneficial, and further research were required to focus on this matter.

## Conclusions

A systematic review and meta-analysis were performed to investigate AE patients' seizure outcome-related factors. The results indicated that onset with seizures, status epilepticus, EEG abnormalities, MRI abnormalities, and longer time from clinical onset to immunotherapy were risk factors for persistent seizures in these patients. The numerous proposed predictors could help to treat physicians formulate prevention strategies for the development of epilepsy. In clinical practice, we should prioritize immunotherapy to control acute seizures as soon as possible and improve seizure outcomes after the acute phase.

## Data availability statement

The original contributions presented in the study are included in the article/supplementary material, further inquiries can be directed to the corresponding author/s.

## Author contributions

WL guided the design of the study and reviewed the manuscript. PL designed the study, screened the literature, extracted data, performed the statistical analysis, and wrote the manuscript. RZ and QC supported the screening of the literature and extracted the data. QC supported the visualization of the results. All authors contributed to the article and approved the submitted version.

## Conflict of interest

The authors declare that the research was conducted in the absence of any commercial or financial relationships that could be construed as a potential conflict of interest.

## Publisher's note

All claims expressed in this article are solely those of the authors and do not necessarily represent those of their affiliated organizations, or those of the publisher, the editors and the reviewers. Any product that may be evaluated in this article, or claim that may be made by its manufacturer, is not guaranteed or endorsed by the publisher.
